# Interoceptive Anxiety and Body Representation in Anorexia Nervosa

**DOI:** 10.3389/fpsyt.2018.00444

**Published:** 2018-09-21

**Authors:** Sahib S. Khalsa, Mahlega S. Hassanpour, Michael Strober, Michelle G. Craske, Armen C. Arevian, Jamie D. Feusner

**Affiliations:** ^1^Laureate Institute for Brain Research, Tulsa, OK, United States; ^2^Oxley College of Health Sciences, University of Tulsa, Tulsa, OK, United States; ^3^Department of Psychiatry and Biobehavioral Sciences, Semel Institute for Neuroscience and Human Behavior, David Geffen School of Medicine, University of California, Los Angeles, Los Angeles, CA, United States; ^4^Department of Psychology, University of California, Los Angeles, Los Angeles, CA, United States

**Keywords:** anxiety, panic, fear, eating disorder, interoception, palpitation, dyspnea, arousal

## Abstract

Individuals with anorexia nervosa (AN) typically display anxious traits prior to the onset of food avoidance and weight loss that characterize the disorder. Meal associated anxiety is an especially common clinical feature in these patients, and heightened sensitivity to sympathetically mediated interoceptive sensations has also been observed. However, it remains unclear how heightened interoceptive sensitivity relates to experiences of anxiety before and after meals. To investigate this relationship, we experimentally induced anxiety and panic symptoms with isoproterenol, a peripheral sympathetic agonist similar to adrenaline, across several different conditions: during panic provocation, during anticipation of a 1,000 Calorie meal, and after meal consumption. Fifteen AN and 15 age- and sex-matched healthy comparisons received bolus infusions of isoproterenol and saline in a double-blinded, randomized design. Participants rated anxiety symptoms after each infusion, completed panic rating scales, and traced the location of perceived palpitations on a manikin to index interoceptive “body map” representation. The AN group reported significantly elevated anxiety relative to healthy comparisons during infusions before and after the meal, but surprisingly, not during panic provocation. These symptoms were accompanied by geographical differences in patterns of perceived heartbeat sensations across each condition. In particular, the AN group localized heartbeat sensations disproportionately to the chest during meal related saline infusions, when no cardiorespiratory modulation actually occurred. The AN group also showed a trend toward higher panic attack rates during the meal anticipation period. Correcting for anxiety levels reported during saline infusions abolished group differences in anxiety change across all conditions, suggesting a significant contribution of anxious traits in AN. The observation of meal related “visceral illusions” provides further evidence that AN is associated with abnormal interoceptive representation of the heartbeat and suggests that meal consumption, particularly when anticipated, preferentially alters the processing of interoception related signals in AN.

## Introduction

Individuals with anorexia nervosa (AN) often exhibit heightened anxiety well before the onset of food avoidance and weight loss that characterize the disorder (1), and anxious traits are frequent precursors to the morbid diagnostic characteristics of AN (2–4). Anxiety is also over-represented in first-degree relatives of affected individuals (5, 6), supporting the idea that anxious processes are intrinsic to the developmental risk architecture of the illness (7, 8). Despite these and other indications of links between anxiety and eating disorders (2, 9–14), there is a paucity of research into the potential pathophysiological mechanisms of anxiogenesis in AN. For example, it is presently unclear to what extent subjective experiences of anxiety reported by individuals with AN are primarily due to anxious traits (that might generate heightened but nonspecific arousal levels), or whether they are exaggerated by aversive anticipation, and if so, whether aversive anticipation is generalized or is selectively modulated by specific contexts such as hypervigilance toward food cues (15, 16), intolerance of uncertainty (11), or discomfort with bodily sensations (17). Furthermore, it is unknown whether anxiety related to sympathetically mediated interoceptive sensations is generally heightened in AN, if it is additionally impacted by anticipatory or after-effects of eating, or both.

In a previous study we demonstrated that pharmacological infusions of isoproterenol altered meal related interoceptive signal processing in AN (18). The general notion was to systematically perturb the observed body state under different contexts, either shortly before or after a calorically dense meal. Isoproterenol evokes transiently increased cardiac and respiratory sensations by stimulating peripheral beta-adrenergic receptors in the heart and lungs, in a fashion similar to adrenaline (19). AN patients reported feeling the isoproterenol induced sensations (particularly dyspnea) more intensely than healthy comparison participants when anticipating a meal, suggesting a heightened sensitivity to interoceptive signals. However, during the saline infusions the patients also reported feeling significantly more sensations of palpitations and dyspnea during the pre-meal period. The fact that no bodily modulation had actually occurred suggested that this was evidence of abnormal interoceptive prediction error signaling (20, 21). According to this model, psychopathology results from a dysregulated ability to adequately sense what is happening in the body resulting a turbulent reference state (i.e., a “noisy baseline”) (22), attentional bias toward threat (23), increased self-related worry, and dysfunctional learning about bodily states over time (24) [for an overview of current models of interoception's role in psychopathology see (25)]. We did not examine in the previous study whether these interoceptive prediction errors permeated other levels of body representation, such as interoceptive localization, or experiences of anxiety. This is important as higher doses of isoproterenol can elicit panic anxiety (26–28), making it difficult to discern whether the physiologically induced arousal leading to subjective feelings of interoception and anxiety can be dissociated from the anxiousness that is part of the AN phenotype.

In the current study we investigated these issues by assessing the impact of meal anticipation and consumption on interoceptively induced anxiety in AN. To conduct this analysis, we examined previously unreported data from our prior study (18) consisting of panic anxiety and body map ratings. We considered three questions: (1) Does the peripheral modulation of interoceptive sensation with isoproterenol disproportionately increase subjective anxiety associated with meal presentation in AN? (2) Is there a greater panic inducing effect of isoproterenol infusions in AN, irrespective of meal proximity? (3) Is there accompanying evidence of abnormal mapping of bodily sensation that would indicate a disturbance of interoceptive representation in AN? In regard to the first question, we predicted that individuals with AN would display greater isoproterenol-induced anxiety during a meal challenge. In regard to the second, we predicted that individuals with AN would display greater panic anxiety than healthy comparisons during a panic provocation task. In regard to the third question, we predicted that there would be group body map differences, but we did not pre-specify the direction or location due to a lack of pre-existing literature on this topic.

## Materials and methods

### Participants

Fifteen individuals with current or lifetime DSM-5 criteria for AN and 15 healthy comparisons (HC) completed the study^1^ (Table 1). Diagnoses were verified by a clinician using the Mini International Neuropsychiatric Interview (MINI version 6.0) (29). We adopted DSM-5 criteria for AN, principally reflecting the removal of the amenorrhea requirement; diagnoses of comorbid disorders were verified using DSM-IV criteria. HCs were also screened for current major psychiatric illnesses with the MINI, and all participants were screened for lifetime neurological, cardiac and respiratory disease. While AN was required to be the primary disorder in terms of clinical severity, we allowed select comorbid diagnoses in order to obtain a sample representative of patients commonly treated in community settings (Table 2) (see Supplementary Methods for further details). To prevent potential interference with the measurement of panic anxiety, we specifically excluded patients with comorbid panic disorder. To ensure they were not in the acute starvation state, participants with AN were required to have a minimum body mass index (BMI) above 17 on the day of testing. The study was approved by the Institutional Review Board of the University of California Los Angeles (UCLA) and the UCLA Clinical and Translational Research Center Review Committee. All participants provided written informed consent and received compensation for their participation.

**Table 1:** Demographics and baseline self-report measures.

**Measure**	**Anorexia nervosa mean (SD) unless specified**	**Healthy comparison mean (SD) unless specified**	***t*-Test (*df* = 28)**	***p***
Age (years)	23.0 (3.8)	22.3 (4.5)	0.48	0.63
Sex (%)	15 females (100%)	15 females (100%)	–	–
Body mass index (BMI)	19.2 (1.6)	22.7 (3.7)	3.27	0.004
Ethnicity (% Caucasian)	60	40	–	–
Education (years)	14.7 (2.5)	14 (2.2)	0.87	0.39
BDI	12.1 (6.9)	2.1 (2.1)	5.42	<0.001
BAI	13.8 (8.3)	3.1 (3.2)	4.63	<0.001
EDE–global score	2.8 (1.4)	–	–	–
Baseline heart rate (bpm)	70.1 (12.4)	70.5 (9.3)	0.10	0.92
CD25–baseline	5.2 (4.6)	4.0 (1.3)	0.99	0.34
CD25–pre meal	5.30 (2.6)	5.3 (2.6)	0.04	0.97
CD25–post meal	7.1 (4.3)	6.2 (3.1)	0.66	0.52

*SD, Standard Deviation; BDI, Beck Depression Inventory; BAI, Beck Anxiety Inventory; EDE, Eating Disorder Examination scale; CD25, Chronotropic Dose 25, isoproterenol dose in micrograms (mcg) required to increase the heart rate by 25 beats per minute (bpm)*.

**Table 2:** Clinical characteristics of participants.

	**Anorexia nervosa mean (SD) unless specified**	**Healthy comparison mean (SD) unless specified**
AN restricting subtype (%)	73.3	0 (0)
Age of onset (years)	16 (2.4)	–
Illness duration (years)	6.8 (4.1)	–
Largest weight loss (lbs)	27.9 (10.2)	–
Lowest BMI	15.3 (1.7)	–
Comorbid secondary diagnoses [*N* (%)]	–	–
GAD	4 (27)	0 (0)
MDD	2 (13)	0 (0)
OCD	2 (13)	0 (0)
>2 comorbid disorders	2 (13)	0 (0)
Psychotropic medication N (%)	4 (27)	0 (0)

*Diagnoses based on DSM-5. GAD, generalized anxiety disorder; MDD, major depressive disorder; OCD, obsessive compulsive disorder*.

### Infusions

Participants received three sets of isoproterenol and saline infusions on the same day.

### Panic provocation assessment

All participants received single blinded bolus intravenous infusions in the same fixed order as: 0.1 micrograms (mcg) → saline → 4.0mcg → saline → 1.0mcg → 2.0mcg → saline [protocol identical to (28)]. We chose this order so that the first several infusions could: (1) rule out the possibility of adverse (i.e., allergic) reactions to isoproterenol, and (2) acclimatize participants to the experimental setup, so that anxiety reported during isoproterenol infusion was related to the experience of isoproterenol-induced sensations rather than anticipatory anxiety induced by infusion administration. We selected the 4.0 mcg bolus dose in order to maximize the likelihood of inducing panic anxiety on the basis of: (1) safety, (2) tolerability, and (3) similar heart rate response to studies using continuous isoproterenol infusions (30). We included lower doses (1.0 and 2.0 mcg) in order to evaluate the degree to which panic responses were dose-related and to establish whether baseline sensitivity to isoproterenol (measured via the chronotropic dose 25 or CD25) differed between groups. CD25, the dose necessary to increase the participant's heart rate by 25 beats per minute above baseline, is a commonly reported measure of peripheral sensitivity to adrenergic sympathetic stimulation (31, 32), and it is known to vary in underweight AN (33). Immediately after each infusion, participants rated the maximum intensity of how “anxious, tense, or nervous” they had felt during the infusion on a visual analog scale. Anxiety ratings could range from 0 (“not at all” or “none”) to 10 (“extremely” or “most intense ever”). They also completed a panic symptom rating scale containing all 13 DSM-5 symptoms of a panic attack (34). To operationalize the experience of panic anxiety we included several variants in terminology (35, 36). Thus, in addition to measuring levels of self-reported anxiety, we indexed fear and panic by having participants rate the terms “scared, fearful or afraid” and “panicked” [criteria were identical to (28)]. Each emotion and panic symptom rating could range from 0 (“not at all” or “none”) to 10 (“extremely” or “most intense ever”), respectively. To define whether panic anxiety had occurred during each infusion, intensity rating increases of 50% or more (i.e., ≥ 5 point increase on the 0 −10 scale) in four or more panic symptoms were required compared with pre-infusion ratings [similar to (26, 27)] (see Supplemental Methods for further information).

### Meal assessment

The second and third infusion sets were linked to an assessment of pre and post meal states. The second infusion set occurred immediately prior to a 1,000 Calorie meal, while the third infusion set occurred immediately upon meal completion. During each pre and post meal infusion set participants received 14 bolus infusions: 7 isoproterenol (0.1, 0.25, 0.5, 0.75, 1.0, 2.0, 4.0 mcg), and 7 saline. Infusion administration was randomized and double blinded per previous protocols from our group (18, 19, 37). Participants rated their experience of internal body sensations during and immediately following bolus infusions. The lowest doses included in these sets (0.1, 0.25, 0.5, 0.75 mcg) were intended to improve the detection of deviations in interoceptive awareness from baseline [as reported in (18)]. They were not expected to induce panic anxiety, and so we do not report on that aspect here.

### Meal protocol

Participants selected two meals (breakfast and lunch) from a menu that provided three different isocaloric meal options. Breakfast was provided in order to standardize the timing between meals. The breakfast calorie content (300 Calories) reflected a standard meal likely to be recommended during regular daily intake, and one that prior research has shown is consumable and less likely to induce severe anxiety in eating disorder participants in a research setting (38, 39). The lunch calorie content (1,000 Calories) reflected an amount expected to be associated with aversive anticipation in individuals with AN, but not so high that it would be unreflective of a typical lunch meal consumed by an unaffected individual. For example, from the lunch menu participants were required to select a dessert option (cheesecake, chocolate covered ice cream bar, or cookie).^2^ Calorie content and calorie consumption for each meal was measured by a registered dietician.

**Figure 1:**
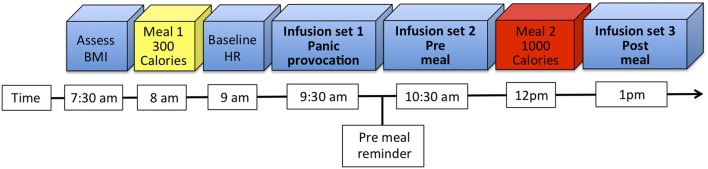
Experimental timeline. Participants arrived at 7:30 a.m. in the fasting state, at which point their body mass index (BMI) was assessed, and a urine sample collected to assess pregnancy and substance use status. After eating breakfast (Meal 1) and filling out several questionnaires, baseline heart rate (HR) was measured. Each infusion set proceeded in order. Participants were reminded about the caloric content and menu for the lunch meal (Meal 2) immediately prior to the second infusion set.

Participants fasted overnight, and ate breakfast upon arrival. Infusions began 90 min afterwards (Figure 1). To maximize food-related emotional arousal, participants were reminded they would break for the 1,000 Calorie lunch immediately prior to the second infusion set, and that they were required to consume the entire meal. The third infusion set started immediately after lunch completion.

### Apparatus

We recorded heart rate continuously during all infusions with an MP150 acquisition unit (Biopac Systems, Inc.).

### Body map assessment

Immediately after each infusion participants were instructed to “draw the areas where you felt your heartbeat (be as accurate as possible)” on a paper manikin (identical to (19, 37)). These outlines were traced manually onto a digital manikin by an expert tracer blinded to the diagnostic grouping [as in (40)]. The digital manikin mobile application was developed and hosted on the Chorus platform (41, 42). Before moving further a reliability check across 90 randomly selected maps, traced onto the digital manikin by a second tracer, yielded an inter-tracer Dice (43) similarity coefficient ≥ 0.77, suggesting quantitatively reliable agreement comparable to other published manual tracing approaches (44). We then calculated proportional body maps from the binary tracings for each group and condition (Supplementary Figure 1), selecting only doses for which all 15 participants generated tracings (saline, 2.0, and 4.0 mcg). Thus, each pixel value in the proportional map was equal to the total number of participants reporting sensation in that pixel (maximum of 15) divided by total number of participants (maximum of 15). We then applied spatial smoothing using a Gaussian kernel with full width half maximum of 6 pixels. This smoothing size was determined from the inter-tracer reliability analysis, representing half of the average non-overlapping areas observed between the two tracers.

### Statistical analysis

Demographic data were analyzed via independent samples *t*-tests. Continuous data were analyzed using general linear models with repeated measures, with group, condition (panic provocation, pre meal, post meal) and dose as fixed effects and individual participants as random effects. In order to differentiate meal related effects, data from the pre meal and post meal infusions were entered into the same analysis. Dichotomous variables were analyzed using the Chi square test. All tests were two tailed, and *p*-values ≤ 0.05 were considered statistically significant.

### Statistical analysis of body maps

To evaluate between-group differences in the body maps, for each pixel, we calculated the test statistic using the z-formula for proportion (45)

$$\begin{array}{l} z=\frac{pAN-pHC}{\sqrt{pTot\left(1-pTot\right)}\;\sqrt{\frac{1}{NAN}+\frac{1}{NHC}}} \end{array}$$

where p_AN_ is the proportion of participants in the AN group who reported having sensation on that pixel and p_HC_ is the same for the HC group, pTot is the proportion of participants having sensation when both groups are combined, and N_AN_ and N_HC_ are the number of participants in the AN and HC groups. To estimate the *p*-value for the calculated z-value, we performed a permutation analysis in Matlab (Mathworks, Inc.). In this analysis, we assumed that under the null hypothesis, the group labeling of participants (AN or HC) is arbitrary, and one can estimate the probability distribution of the test statistic under the null hypothesis by relabeling participants many times and computing the test statistic. When participants are independent from one another, the total number of possible relabelings would be (N_AN_+N_HC_)!/(N_AN_! N_HC_!). We used 5,000 relabelings (*N*_*res*_, randomly selected from all the possible relabelings) which resulted in an acceptable precision error for the *p*-value estimation (<0.0001) via a random permutation or Monte-Carlo permutation test procedure [modeled after (46)]. The *p*-value for each pixel was subsequently calculated by computing the number of occurrences of z-value in the resampled conditions equal or larger than the *z*-value of the actual sample. Pixels with *p*-values ≤ 0.05 were considered to have significantly more hits (i.e., reported to have sensation) by group AN compared to group HC (47) (see Supplementary Methods for further details).

## Results

### Heart rate response

Both groups exhibited similar resting heart rates at baseline [*t*_(28)_ = 0.10, *p* = 0.92] (Table 1). While the bolus isoproterenol infusions elicited significant dose-dependent responses across each condition, there were no significant group differences across any of the conditions (Supplementary Figure 2, see Supplementary Results).

### Meal consumption

During screening and eligibility assessment, several potential participants refused to enroll in the study since they were uncomfortable with the amount of calories in the lunch meal. Of the individuals who consented to participate in the study, they all consumed the breakfast meal in entirety, and there were no significant group differences in lunch calories consumed between groups [*F*_(1, 28)_ = 1.48, *p* = 0.24](see Supplementary Results for further details).

### Anxiety ratings

**Figure 2:**
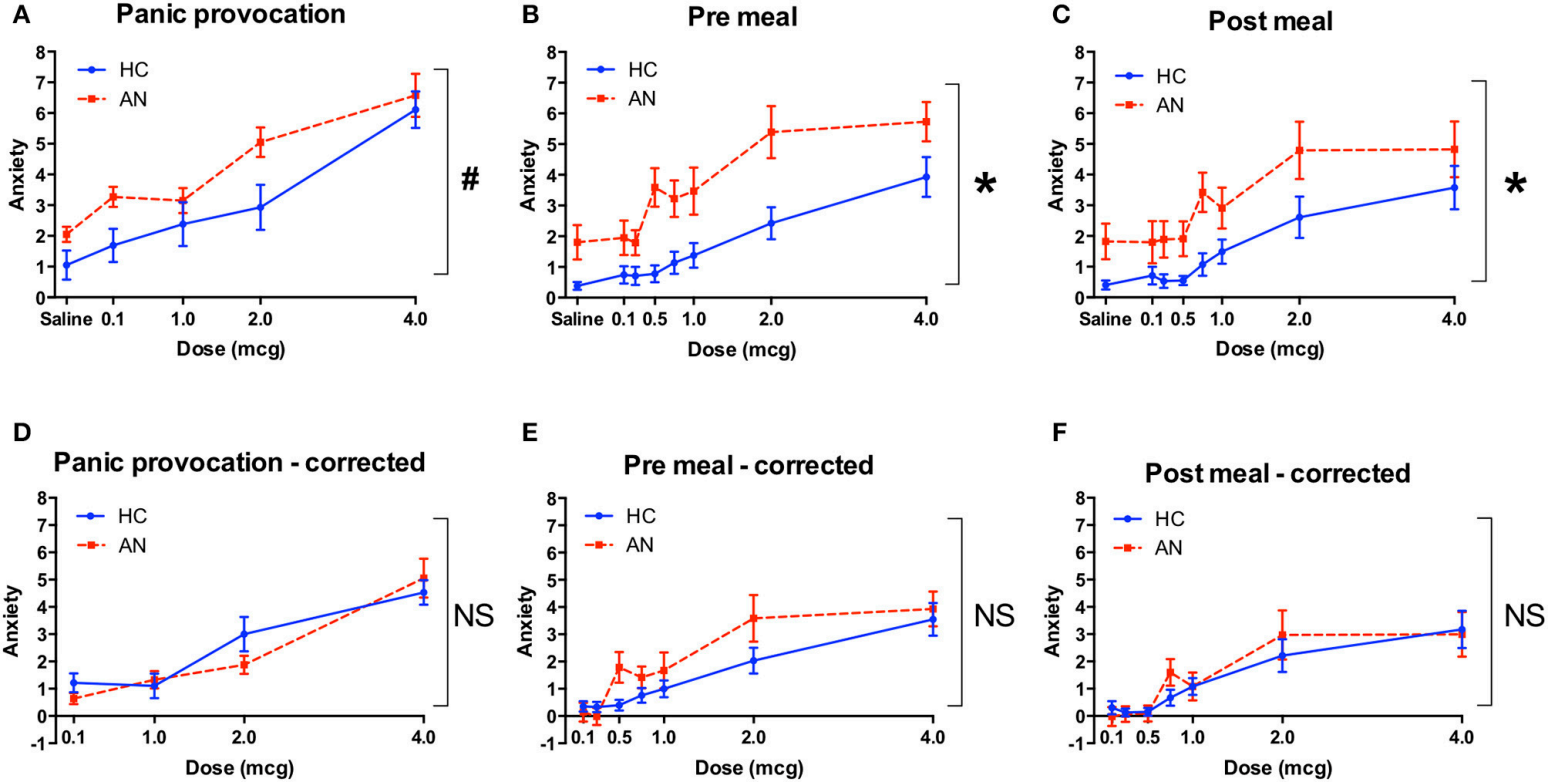
Retrospective anxiety ratings during isoproterenol infusion. **(A)** Anxiety during panic provocation condition. The group effect showed a trend toward significance (^#^*p* = 0.08). **(B)** Anxiety during pre meal infusion, prior to consuming a 1000 Calorie meal. There was a significant group effect (^*^p = 0.009). **(C)** Anxiety during post meal infusion, after consuming a 1,000 Calorie meal. There was a significant group effect (^*^p = 0.009). **(D)** Corrected anxiety during panic provocation. Anxiety was baseline corrected by subtracting the average anxiety reported during saline infusions from the anxiety reported at each dose. There were no group differences in anxiety during the panic provocation **(D)**, pre meal **(E)**, or post meal **(F)** conditions. Axis markers for the 0.25 and 0.75 mcg doses are omitted to improve figure clarity. AN, anorexia nervosa; HC, healthy comparison; NS, non-significant.

The AN group reported greater levels of anxiety at baseline, prior to starting the panic and meal infusion conditions [*F*_(1, 28)_ = 6.4, *p* = 0.018]. They also reported a trend toward higher levels of isoproterenol-induced anxiety during the panic condition [*F*_(1, 28)_ = 3.3, *p* = 0.08) (Figure 2A), and significantly higher anxiety across meal conditions [*F*_(1, 28)_ = 7.8, *p* = 0.009] (Figures 2B,C). However, after controlling for anxiety experienced during saline infusions, there were no longer any group differences observed in the magnitude of anxiety increase from low to high doses. This was true for the panic condition [*F*_(1, 28)_ = 0.02, *p* = 0.89] (Figure 2D) and across meal conditions [*F*_(1, 28)_ = 0.75, *p* = 0.39] (Figures 2E,F). There were also no significant group by dose interactions for anxiety in the panic provocation condition, under either uncorrected [*F*_(1, 3)_ = 1.99, *p* = 0.12] or corrected [*F*_(1, 3)_ = 1.73, *p* = 0.17] conditions. Similarly, for the meal condition there were no significant group by meal interactions for anxiety under either uncorrected [*F*_(1, 1)_ = 1.3, *p* = 0.26] or corrected [*F*_(1, 1)_ = 0.76, *p* = 0.39] conditions. There were no significant three-way interactions (group × meal × dose) for isoproterenol induced anxiety under either uncorrected [*F*_(1, 6)_ = 1.64, *p* = 0.18] or corrected [*F*_(1, 6)_ = 1.21, *p* = 0.30] conditions. These results indicate that while anxiety levels were higher in the AN group overall during the meal related conditions, the amount of change in subjectively experienced anxiety from low to high doses was similar for both groups.

### Panic rates

**Figure 3:**
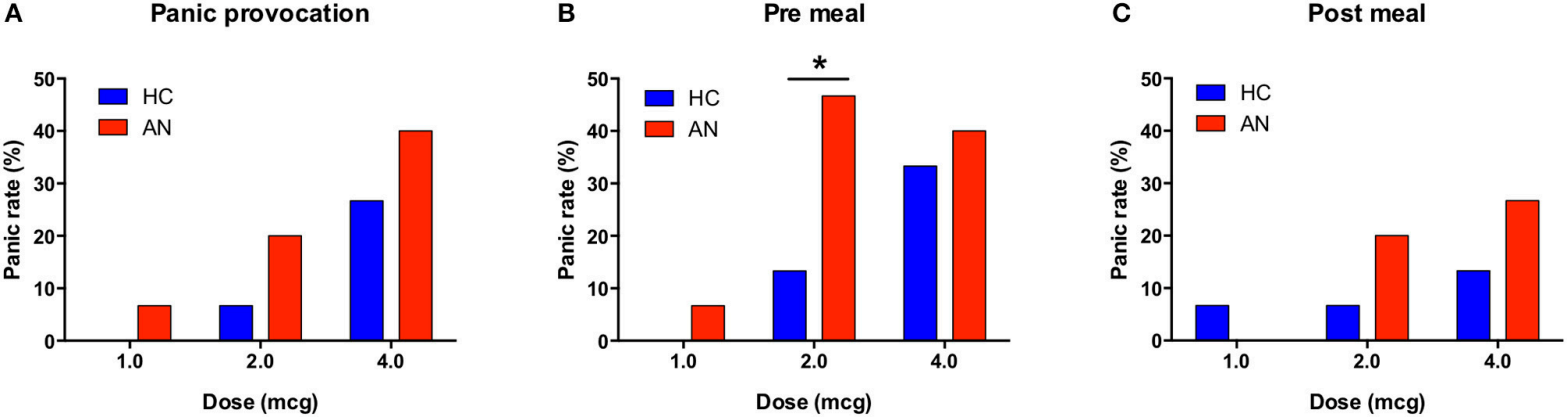
Panic attack rates during bolus isoproterenol infusions. **(A)** For the panic provocation condition, there was no significant difference in the panic rate across all doses (*p* = 0.18) or at the individual dose level. **(B)** For the pre meal condition, there was a trend toward a higher panic rate in AN across all doses (*p* = 0.08), and there was a significantly higher panic rate at the 2.0 mcg dose (*p* = 0.013). **(C)** For the post meal condition, there was no significant difference in panic rate across all doses (*p* = 0.24) or at the individual dose level.

There were no significant group differences in the overall rate of panic anxiety for panic provocation [(χ2_(1)_ = 1.79, *p* = 0.18], the pre meal [(χ2_(1)_ = 3.07, *p* = 0.08], or post meal condition [χ2_(1)_ = 1.37, *p* = 0.24]. Given the trend toward significance in the pre meal condition, and our intention to evaluate for meal related effects, we examined whether there was evidence of a differential panic rate at individual doses. We found that a significantly greater proportion of the AN group panicked during the pre meal condition at the 2 mcg dose [χ2_(1)_ = 3.96, *p* = 0.046] (Figure 3).

### Interoceptive representation

**Figure 4:**
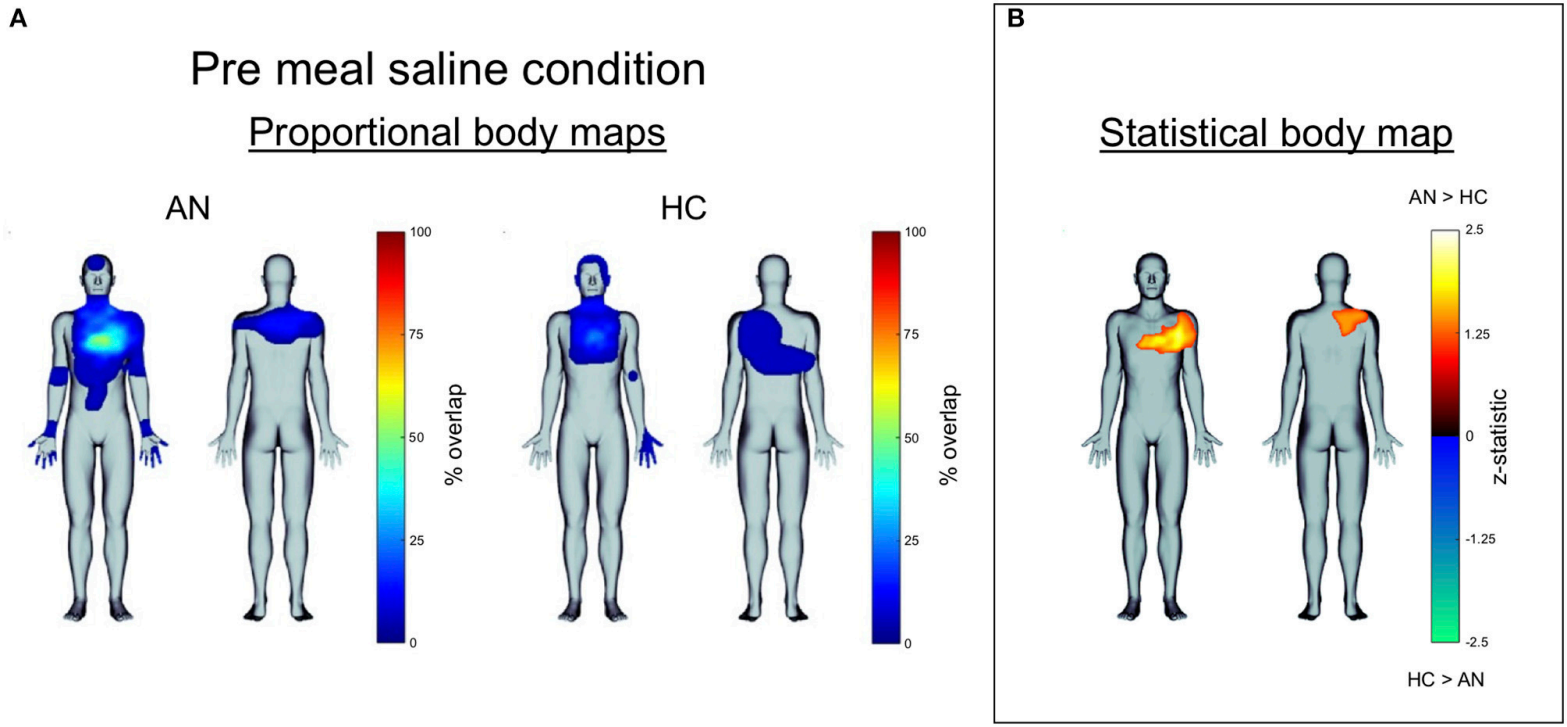
**(A)** Proportional body map showing group differences in heartbeat sensation topography during the pre meal infusions of saline. The AN group showed a higher percent overlap in the chest as well as heartbeat sensations in the upper abdomen and extremities. **(B)** Statistical body map (thresholded at *p* ≤ 0.05) for the same condition showing that only a subset of these regions reached statistical significance: the AN group reported feeling heartbeat sensations more frequently in the upper left chest and upper right back during pre meal infusions of saline.

**Figure 5:**
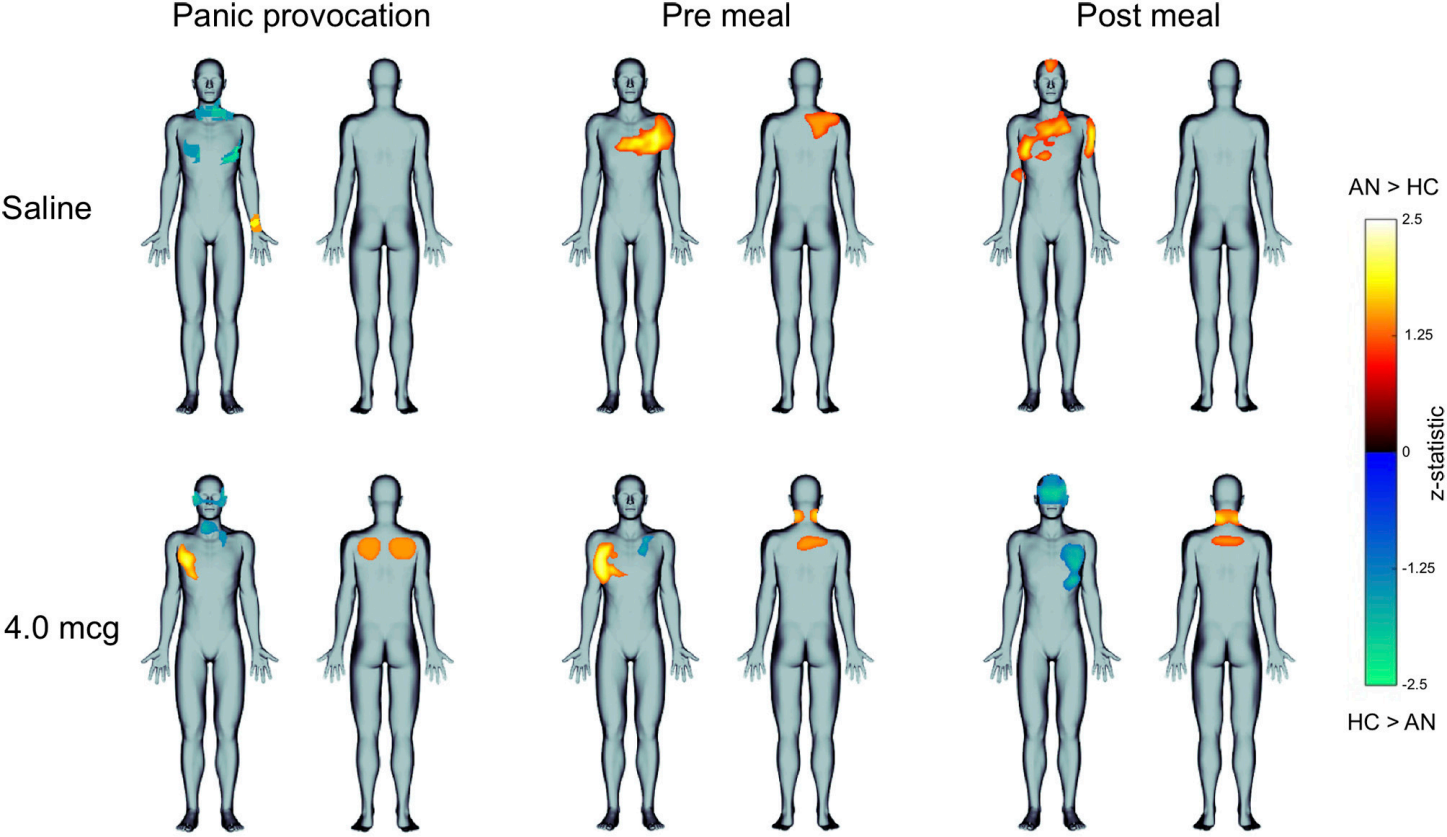
Statistical body maps (thresholded at *p* ≤ 0.05) showing quantitative group differences in heartbeat sensation localization during the panic provocation, pre meal, and post meal conditions. **(Top)** During the pre and post meal infusions of saline the AN group reported feeling heartbeat sensations more frequently in different regions of the chest, when no interoceptive perturbations were delivered, whereas during panic provocation saline infusions the HC group reported feeling heartbeat sensations more frequently in the inferolateral chest and anterior neck. **(Bottom)** During the 4 mcg perturbations, when similar physiological responses were observed in both groups, the groups showed opposing patterns: the AN group showed reported feeling heartbeat sensations more frequently in the right side of the chest during panic and pre meal infusions, whereas the HC group reported feeling heartbeat sensations more frequently in the left chest and upper face during the post meal infusion. For brevity, only results from the saline and 4 mcg dose are displayed.

We observed qualitative group differences in the topographies of heartbeat sensation localization, as reflected by the proportional body map distributions (Figure 4A shows an example for the pre meal saline condition). These differences were confirmed by quantitative differences in the statistical body maps (Figure 4B). Inspection of all statistical body maps revealed different patterns in the geographical localization of heartbeat sensation body maps across each condition (Figure 5). During the saline infusions, when no exogenous cardiorespiratory perturbation occurred, the HC group reported more sensation in the inferolateral chest and neck in the panic provocation condition, whereas during the meal conditions the AN group reported disproportionately greater sensations in the superolateral chest, upper abdomen, upper back, and vertex of the head. During the 4 mcg infusions, when similar physiological responses were observed in both groups, the HC group preferentially reported heartbeat sensations in the neck, face and left lateral chest across conditions, whereas the AN group preferentially reported heartbeat sensations in the right lateral chest, posterior neck, and upper back predominantly in the meal conditions (especially pre meal).

### Exploratory analysis: pre vs. post meal anxiety

**Figure 6:**
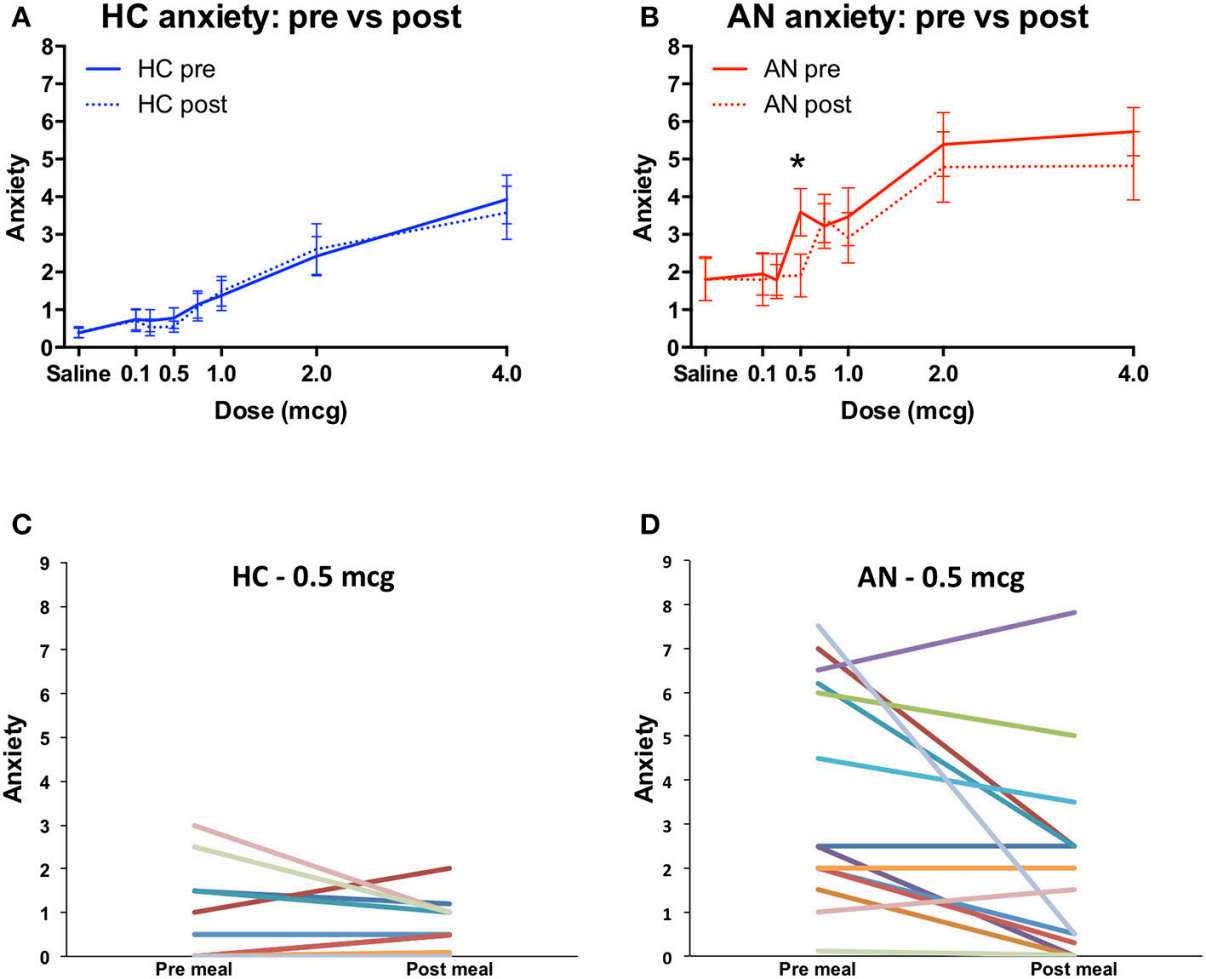
Pre vs. post meal anxiety. **(A)** Healthy comparisons (HC). **(B)** Anorexia nervosa (AN). The AN group reported significantly higher pre meal anxiety during the 0.5 mcg dose (^*^p = 0.026, uncorrected). These first two data sets are identical to Figures 2B,C, and are shown superimposed here to better illustrate the within group differences in meal related anxiety exhibited by the AN group. **(C)** Individual pre vs. post meal anxiety changes at the 0.5 microgram dose for healthy comparisons and **(D)** anorexia nervosa. At this dose most HC participants reported no anxiety during both time points, whereas most AN participants reported anxiety.

Since the AN group showed evidence of heightened meal associated anxiety consistent with our a priori hypothesis, we conducted an exploratory analysis to examine whether there were differences in pre and post meal anxiety at the level of individual doses. We observed a general pattern of decreased post meal anxiety only in the AN group (Figures 6A,B). At the individual dose level the AN group showed a statistically significant reduction in post meal anxiety at 0.5 mcg [t_(28)_ = 2.44, *p* = 0.026, two tailed, uncorrected]. Plotting individual anxiety reductions at this dose revealed that the majority of AN participants (10 out of 15) reported lower post meal anxiety, with a minority showing no change (3 out of 15) or an increase in anxiety (2 out of 15) (Figure 6C). Only a minority of HCs reported lower post meal anxiety (3 out of 15) whereas the majority showed no anxiety change (9 out of 15), or an increase (3 out of 15) (Figure 6D).

## Discussion

In the current study of AN we experimentally manipulated interoception related anxiety and panic symptoms in the same context as meal consumption. There were several important findings related to our hypotheses: (1) Contrary to our hypothesis the relative change in anxiety from low to high doses was similar for both groups. The absolute levels of interoceptively induced anxiety were higher in the AN group across the meal period, suggesting a backdrop of heightened meal related anxiety expression in the AN group. (2) Also contrary to our prediction, the AN group failed to show a greater panicogenic sensitivity to isoproterenol infusions during non-meal related panic provocation. Instead, there was a trend toward greater panicogenic effect of isoproterenol infusions in AN during meal anticipation (significant at the 2.0 mcg dose), supporting the possibility that meal anticipation represents a specific temporal window of heightened interoception related anxiety susceptibility in AN. (3) Using body maps to index interoceptive representation, we found that the AN group showed discrepancies in the geographical localization of heartbeat sensation, suggesting the presence of abnormal interoceptive body representation. We consider the implications of these findings in further detail below.

Consistent with many other studies of AN which have observed higher trait anxiety levels (2, 9, 10) we found that the AN group reported heightened anxiety across multiple contexts: in self report scales at study entry (i.e., Beck Anxiety Inventory), during baseline visual analog scale ratings prior to receiving any infusions, and during intravenous infusions in which no physiological changes were induced (i.e., saline). This finding was unsurprising. The observation that both groups reported similar increases in anxiety from low to high doses after accounting for these baseline differences was surprising, and went against our predictions. It underscores the degree to which nonspecific dimensional factors (perhaps such as anxious temperament, hypervigilance, discomfort with bodily sensations, and weight gain induced fear) might contribute to baseline reports of heightened anxiety in clinical settings. For example, in the current study patients in the AN group reported on average experiencing anxiety at intensity levels of approximately 2 out of 10 across all saline infusion conditions, compared with the HC group's reported anxiety intensity level of approximately 0.5 out of 10. Given the similar increases in interoceptively induced anxiety across both groups, we speculate that interventions capable of reducing these baseline levels of heightened anxiety could potentially buffer patients from experiencing higher situational anxiety (as in the case of meal onset periods).

In an exploratory analysis we observed some evidence of attenuated anxiety in the AN group during exposure to low doses of isoproterenol (particularly 0.5 mcg) from pre to post meal time periods. Although this exploratory finding needs to be confirmed in a separate study, we believe that any observation of a reduction in anxiety across the meal period in AN warrants attention. For example, the attenuated response could relate to the development of affective tolerance to the drug due to a weakened stimulus-response pairing (i.e., habituation), or alternatively, anxiety relief associated with removal of exposure to food, an anxiogenic stimulus (i.e., negative reinforcement). Additional experiments could determine which of these two possibilities was most likely. This is important because pre meal anxiety poses a frequent challenge in AN treatment settings: it is associated with lower caloric intake and has been proposed as a potentially important therapeutic target (38). Unfortunately, typically effective anxiolytic medications such as alprazolam are ineffective in individuals with AN, and they do not increase caloric intake (48). In contrast, cognitive behavioral therapies for AN utilizing exposure therapy can promote food intake (49) and increase weight in AN (50), although the gains are modest and are marked by high dropout rates. Limited responses to exposure therapy using food may be related to the fact that this treatment requires AN patients to voluntarily engage in repeated and sometimes prolonged confrontation with the primary stimulus that disproportionately provokes their fear. As a consequence, this type of approach may be too aversive for many individuals to tolerate, at least in the early phases of treatment. Recent psychotherapeutic treatments have instead targeted reinforcing or maintaining factors in treatment, such as family based interactions around food (51), anxious temperament (3), reward processing (52, 53), and habit training (54). The current study adds to this debate by considering whether interoceptive interventions to attenuate anxiety during meal anticipation might warrant further investigation.

Our observation that heartbeat sensations were felt to be localized in discrete body regions in the absence of cardiorespiratory stimulation suggests the presence of “visceral illusions” and provides further evidence that AN is associated with abnormal meal related interoceptive representation of the heartbeat. Although we have previously characterized the bodily localization of isoproterenol-induced heartbeat sensation in healthy and brain lesioned individuals (19, 37, 40), the current study demonstrates quantitative group differences in the mapping of these interoceptive representations in a sample of psychiatric patients. The finding that patients preferentially localized illusory sensations to the left side of the chest during meals in the absence of sympathetic stimulation has several interpretations. It could be caused by source misattribution (i.e., a mistaken belief that the heart is predominantly located on the left), or alternatively, could reflect the presence of interoceptive prediction errors as we have previously suggested (18). The cause of the AN group's tendency to localize sensations to the back and lateral aspect of the right chest during actual sympathetic stimulation, irrespective of condition, is also unclear. Since individuals with lower BMI have been observed to display greater cardiac interoception under resting conditions (55, 56), one potential mechanism could be greater reverberation of myocontractile force throughout the body due to the lower BMI in the AN group. Another mechanism could be due to illness characteristics, such as elevated anxiety or eating disorder symptoms, though this is speculative. To evaluate the role of illness characteristics a more clinically relevant question would need to be addressed. Namely, does the presence or absence of reported somatic sensations in specific body regions help predict if individuals are anxious, exhibit low BMI, or eating disorder symptoms? Answering this question would require a different approach and likely a larger sample than was possible in the present study. Nevertheless, the current results could be considered as a proof-of-principle demonstrating the feasibility of interoceptive body map quantification at the group level. It is worth noting that this quantitative body mapping technique shares some general similarities with other recent approaches (46, 57) in the sense that it employed a graphical interface and a virtual avatar. However, two fundamental differences relate to our use of a physiological probe to elicit interoceptive sensation, and a binary mapping procedure to detect the presence/absence of sensation across the entire body surface (front and back).

The current study examined the impact of two specific probes relevant to the experience of anxiety in AN: physiological perturbation with a sympathetic agonist, and manipulation of expectations surrounding consumption of a relatively large meal. By examining the anxiogenic influences of these probes separately, we identified evidence (preliminary, given that findings were at trend level and the sample size was small) of a synergistic influence of pre meal time periods on the anxious response to an interoceptive perturbation. This is clinically informative because it suggests that the anticipation of a meal constitutes a time when AN patients have a disproportionate vulnerability to the subjective effects of physical triggers of anxiety and panic symptoms. This subjective vulnerability could not be caused by different physiological reactions to the drug, as both groups showed nearly identical responses.

The current results are relevant to cognitive-behavioral theories of the etiology of anxiety. For example, the observed trend for heightened pre meal panic could be driven solely by increased sensitivity to sympathetically-mediated interoceptive cues, solely by cognitive cues signaling meal onset, or by a combination of these two factors. In each case these responses would be speculated to occur within discrete sectors of the central nervous system, for example in the insular cortex or amygdala in the case of heightened interoceptive sensitivity (28, 40, 58–60), and/or in the prefrontal cortical and striatal regions in the case of cognitive processing cues (59, 61). Our prior observation of abnormally intense isoproterenol-induced sensations of dyspnea in AN during meal anticipation (18), and recent findings of abnormal insula activation during respiratory stimulation in women remitted from AN (62) suggest that erroneous processing of interoceptive respiratory cues is a key contributing factor. While we cannot rule out an additional role for cognitive processing from these studies, future work incorporating parametric designs (e.g., high/low interoceptive/cognitive loading) could help distinguish individuals with disproportionate sensitivity to each process, leading eventually to personalized interventions. For example, knowing whether the source of anxiety in a particular AN individual comes from sensitivity to interoceptive vs. meal related (cognitive) cues would help to develop a personalized medicine approach. In this type of treatment, targeting the mechanisms underlying an individual's pathology (e.g., interoceptive vs. cognitive vs. both) might lead to more specific and effective improvement in symptoms. From a practical standpoint, if the process of anticipating a meal induces illusory and aversive bodily perceptions in AN individuals, then training them to be aware and non-reactive to such illusory signals might better allow them to focus on ongoing rather than anticipated sensory signals. Whether this type of intervention would concomitantly reduce anxiety awaits further investigation. The current study helps by laying the groundwork for such future interventions.

### Limitations and future considerations

The current study has several limitations. (1) The small sample size effectively prevented examination of potentially relevant covariates, such as the effects of medication status and co-occurring disorders. Replicating this experiment in a larger sample would help to increase confidence in the observed findings. However, the approach to separating panic related effects from meal related effects across multiple features of interoception can be viewed as a form of deep phenotyping (63) capable of yielding multiple novel insights. (2) Since we purposefully excluded patients with comorbid panic disorder in an effort to avoid introducing a non-specific bias in response to isoproterenol infusions, we cannot address whether or not the effects may be greater in this subgroup, or may have been greater had we not excluded this subgroup. Both outcomes seem likely as isoproterenol and other pharmacological perturbations have well described effects on triggering panic anxiety in panic disorder (64–66). However, had we included such patients we may have seen group differences in anxiety expression during the panic provocation condition that were driven by a non-primary disorder. One way to address the issues of comorbidity would be to recruit an anxious reference sample (e.g., individuals with generalized anxiety disorder) and compare their responses with the AN group. An alternative approach using the RDoC framework (67) would be to compare responses to isoproterenol infusions and meal challenges in different sets of groups across transdiagnostic dimensions of disturbed eating behaviors and anxiety sensitivity. (3) Panic provocation always occurred pre meal (rather than in randomized or counterbalanced order), and thus it could have been potentially confounded by some degree of meal anticipation. Nevertheless, the fact that panic rates were not higher in the AN group during panic provocation suggests that perhaps it was not proximal enough to the meal to have appreciable effects. (4) The current study did not separately examine the impact of meal related anxiety (i.e., meal anticipation without infusions), and therefore, was unable to dissociate anticipatory anxiety linked to meals from the heightened trait anxiety generally observed in AN individuals. (5) Due to a long day of data collection interoceptive traits were not measured in this study, preventing the examination of links between such traits and the current findings.

## Conclusion

In summary, the current study provides evidence that meal related anxiety in AN also encompasses interoceptive processes, and that AN is marked by meal related differences in the bodily mapping of interoceptive sensations.

## Disclosure

AA is founder of Insight Health Systems, Arevian Technologies Inc., and Open Science Initiative.

## Author contributions

SK, MC, and JF designed the research. SK and JF performed the research. SK, MH, and AA analyzed the data. SK, MH, MS, MC, AA, and JF wrote the paper.

### Conflict of interest statement

The authors declare that the research was conducted in the absence of any commercial or financial relationships that could be construed as a potential conflict of interest.

## Footnotes

^1^Two HC participants were excluded due to incomplete data and two AN participants were excluded due to abnormal electrocardiogram changes during the study. This study was not registered as a clinical trial as the data were collected prior to the updated guidelines on categorization of clinical trials. Accordingly, a CONSORT diagram is unavailable for the study.

^2^Participants were informed that regardless of the meal option selected, breakfast contained 300 Calories and lunch contained 1000 Calories.
