# Supplementary material for: Interoceptive Anxiety and Body Representation in Anorexia Nervosa
Source: Front Psychiatry. 2018 Sep 21;9:444. doi: 10.3389/fpsyt.2018.00444 (PMC6160545; doi:10.3389/fpsyt.2018.00444)
Supplement: Supplementary file 1 [file Data_Sheet_1.docx]

**Interoceptive anxiety and body representation in anorexia nervosa**

Sahib S. Khalsa, Mahlega S. Hassanpour, Michael Strober, Michelle G. Craske,

Armen C. Arevian, and Jamie D. Feusner

**Supplementary materials and methods:**

*Participants*

Diagnoses were verified by a clinician using the Mini International Neuropsychiatric Interview (MINI version 6.0) [1]. We adopted DSM-5 criteria for AN, principally reflecting the removal of the amenorrhea requirement; all other diagnoses were verified using DSM-IV criteria. HCs were also screened for current major psychiatric illnesses with the MINI, and all participants were screened for lifetime neurological, cardiac and respiratory disease. We also allowed patients into the study if they were taking selected medication classes: selective serotonin reuptake inhibitors (SSRI), selective norepinephrine reuptake inhibitors (SNRI), or non-benzodiazepine anxiolytics, provided the dose was stable for ≥ 2 months. These medications were not expected to have a significant impact on interoceptive cue discrimination [2].

*Panic provocation assessment*

All participants received single blinded bolus intravenous infusions in the same fixed order as follows: 0.1 micrograms (mcg)🡪saline🡪4.0 mcg🡪saline🡪1.0 mcg🡪2.0 mcg🡪saline (identical to [3]). We chose this order so that the first several infusions could: (1) rule out the possibility of adverse (i.e., allergic) reactions to isoproterenol, and (2) acclimatize participants to the experimental setup, so that anxiety reported during isoproterenol infusion was related to the experience of isoproterenol-induced sensations rather than anticipatory anxiety induced by infusion administration. We selected the 4.0 mcg bolus dose in order to maximize the likelihood of inducing panic anxiety on the basis of: (1) safety, (2) tolerability, and (3) similar heart rate response to studies using continuous isoproterenol infusions [4].We purposefully included lower doses (1.0 and 2.0 mcg) in order to (1) evaluate the degree to which panic responses were dose-related and (2) establish whether baseline sensitivity to isoproterenol differed between groups (measured via the chronotropic dose 25 or CD25) differed between groups. CD25, the dose necessary to increase the participant's heart rate by 25 beats per minute above baseline, is a commonly reported measure of peripheral sensitivity to adrenergic sympathetic stimulation [5, 6], and it is known to vary in underweight AN [7].

Immediately after each infusion, participants rated the maximum intensity of how anxious, tense, or nervous they had felt during the infusion. They also completed a panic symptom rating scale containing all 13 DSM-5 symptoms of a panic attack [8]. To operationalize the experience of panic anxiety we included several variants in terminology [9, 10]. Thus, in addition to measuring levels of self-reported anxiety, we indexed fear and panic by having participants rate the terms “scared, fearful or afraid” and “panicked” (criteria were identical to [3]). To avoid implicitly priming participants towards these variants, we assessed levels of changes in other emotions, both positive and negative, that we did not expect to be altered by isoproterenol (e.g. “angry, irritated or mad,” “disgusted, grossed out or repulsed,” and “happy, excited or euphoric”). Each emotion and panic symptom rating could range from 0 (“not at all” or “none”) to 10 (“extremely” or “most intense ever”), respectively. To define whether panic anxiety had occurred during each infusion, intensity rating increases of 50% or more (i.e., ≥ 5 point increase on the 0 –10 scale) in four or more panic symptoms were required compared with pre-infusion ratings (similar to [11, 12]).

*Meal protocol*

After providing informed consent participants were asked to select two meals (breakfast and lunch) from a menu that provided three different isocaloric meal options. Participants were required to select a dessert option from the lunch menu (cheesecake, chocolate covered ice cream bar, or cookie). They were informed that regardless of the meal option selected, breakfast contained 300 Calories and lunch contained 1000 Calories. Breakfast was provided in order to standardize the duration of time across participants between the lunch meal experimental condition and the previously eaten meal. The breakfast calorie content was chosen to reflect a standard meal likely to be recommended during regular daily intake, and one that prior research has shown is likely consumable and less likely to induce severe anxiety in eating disorder participants in a research setting [13, 14]. The lunch calorie content was chosen to reflect an amount of food that was expected to be associated with aversive anticipation and an increased affective response in a sample of individuals with AN, but not so high that it would be unreflective of a typical lunch meal consumed by a healthy individual unaffected by AN. Calorie content and calorie consumption for each meal was measured by a registered dietician.

Participants fasted overnight. Upon arrival, they ate breakfast. Infusions began approximately 90 minutes afterwards (Figure 1). To maximize food-related emotional arousal, immediately prior to the second infusion set participants were reminded they would break for the 1000 Calorie lunch that they had selected directly afterwards, and that they were required to consume the full meal. The third infusion set started immediately following lunch completion. Participants were notified simply that the rationale for this procedure was to ensure equal meal consumption status across all participants. Participants were allowed to exit the study if they felt unable to comply with this requirement. During the consent process one individual refused to participate on learning of the caloric quantity of the meals. During the recruitment process several other outpatients noted this food quantity to be a barrier preventing them from study participation. This number was not recorded.

*Statistical analysis of body maps*

Since the saline infusion was the only dose that was repeated (seven times during the pre meal and seven times during the post meal infusions), participants could localize sensations to different regions of the body during different trials. In such cases, in order to identify a single saline map for the permutation analysis, we adopted an inclusive approach whereby all unique areas rated across all saline were collapsed into a single binary map, even if they were only rated once. Due to the number of proportional tests conducted at each dose we considered addressing the multiple comparison problem by calculating the probability distribution for the maximal statistic in the maps [15]. However, we decided against this given the reduced sample size, numerous repeated measures, and exploratory nature of the current study. Our approach for multiple comparison correction was instead as follows: within the permutation/relabeling procedure, we estimated the probability of having maxima equal or larger than the maximum statistic in the actual sample, and if it was less than α = 0.05, we adjusted the pixel level threshold to the (c+1)^th^ maximal statistic where c was α$N_{res}$ [15].

**Supplementary Results**

*Heart rate response*

Both groups exhibited similar resting heart rates at baseline (t(28)=0.10, p=0.92. As expected, bolus isoproterenol infusions elicited significant dose-dependent increases in heart rate across the panic provocation (F(1,3)= 136.4, p<0.0001) and meal conditions (F(1,6)=104.9, p<0.0001). However, the overall heart rate changes did not differ between the groups during the panic provocation (F(1, 28)=0.37, p=0.55) or meal conditions (F(1,28)=1.68, p=0.21) (**Supplementary Figure 2**). There were also no group by dose interactions in the panic condition (F(1,3)=0.10, p=0.96) and no group by pre or post meal interactions (F(1,28) = 1.71, p=0.20), suggesting that the observed physiological changes were equivalent between groups.

*Meal consumption*

All participants consumed the 300 Calorie breakfast in entirety, and all HC participants consumed the lunch meal in entirety. Among the AN group, whereas all but four AN participants consumed the lunch meal in entirety, only one ate a noticeably smaller amount (400 Calories); the other three consumed the majority of the meal (907, 926, and 977 Calories respectively). Despite this individual variability, there were no significant group differences in lunch calories consumed between groups (F(1,28) = 1.48, p = .24).

**Supplementary Figure 1**. (Top) Example body map ratings of the location of felt heartbeat sensation shown for several representative subjects from each group. The numbers in parentheses are schematic, and are intended to illustrate the binary nature of each body map (i.e., sensation is either present or absent at each pixel), with different spatial distributions on the body. (Bottom) Example of a single group proportional body map derived from the summation of each individual body map rating, after masking any tracings located outside of the body, and after applying a 6 pixel FWHM spatial smoothing as described in the methods. Statistical maps were then directly derived from proportional maps after using a permutation test to determine the appropriate threshold for the z-statistic, for each condition during saline and isoproterenol infusions.

**Supplementary Figure 2.** Heart rate responses during isoproterenol infusion. (A) Panic provocation condition. (B) Pre meal condition. (C) Post meal condition. There were no significant group differences or interactions in any condition.

**Supplementary References**

1. Sheehan, D.V., Y. Lecrubier, K.H. Sheehan, P. Amorim, J. Janavs, E. Weiller, T. Hergueta, R. Baker, and G.C. Dunbar, *The Mini-International Neuropsychiatric Interview (M.I.N.I.): the development and validation of a structured diagnostic psychiatric interview for DSM-IV and ICD-10.* J Clin Psychiatry, 1998. **59 Suppl 20**: p. 22-33;quiz 34-57.

2. Marona-Lewicka, D. and D.E. Nichols, *Drug discrimination studies of the interoceptive cues produced by selective serotonin uptake inhibitors and selective serotonin releasing agents.* Psychopharmacology (Berl), 1998. **138**(1): p. 67-75.

3. Khalsa, S.S., J.S. Feinstein, W. Li, J.D. Feusner, R. Adolphs, and R. Hurlemann, *Panic Anxiety in Humans with Bilateral Amygdala Lesions: Pharmacological Induction via Cardiorespiratory Interoceptive Pathways.* J Neurosci, 2016. **36**(12): p. 3559-66.

4. Nesse, R.M., O.G. Cameron, G.C. Curtis, D.S. McCann, and M.J. Huber-Smith, *Adrenergic function in patients with panic anxiety.* Arch Gen Psychiatry, 1984. **41**(8): p. 771-6.

5. Arnold, J.M. and D.G. McDevitt, *Heart rate and blood pressure responses to intravenous boluses of isoprenaline in the presence of propranolol, practolol and atropine.* Br J Clin Pharmacol, 1983. **16**(2): p. 175-84.

6. Mills, P.J., J.E. Dimsdale, S. Ancoli-Israel, J. Clausen, and J.S. Loredo, *The effects of hypoxia and sleep apnea on isoproterenol sensitivity.* Sleep, 1998. **21**(7): p. 731-5.

7. Kaye, W.H., D.T. George, H.E. Gwirtsman, D.C. Jimerson, D.S. Goldstein, M.H. Ebert, and C.R. Lake, *Isoproterenol infusion test in anorexia nervosa: assessment of pre- and post-beta-noradrenergic receptor activity.* Psychopharmacol Bull, 1990. **26**(3): p. 355-9.

8. Craske, M.G., K. Kircanski, A. Epstein, H.-U. Wittchen, D.S. Pine, R. Lewis-Fernández, and D. Hinton, *Panic disorder: a review of DSM-IV panic disorder and proposals for DSM-V.* Depression and Anxiety, 2010. **27**(2): p. 93-112.

9. Adolphs, R., *The biology of fear.* Curr Biol, 2013. **23**(2): p. R79-93.

10. LeDoux, J.E., *The slippery slope of fear.* Trends Cogn Sci, 2013. **17**(4): p. 155-6.

11. Balon, R., R. Pohl, V.K. Yeragani, J.M. Rainey, and P. Weinberg, *Lactate- and Isoproterenol-Induced Panic Attacks in Panic Disorder Patients and Controls.* Psychiatry Res, 1988. **23**: p. 153-160.

12. Pohl, R., V.K. Yeragani, R. Balon, J.M. Rainey, H. Lycaki, A. Ortiz, R. Berchou, and P. Weinberg, *Isoproterenol-induced panic attacks.* Biol Psychiatry, 1988. **24**(8): p. 891-902.

13. Steinglass, J.E., R. Sysko, L. Mayer, L.A. Berner, J. Schebendach, Y. Wang, H. Chen, A.M. Albano, H.B. Simpson, and B.T. Walsh, *Pre-meal anxiety and food intake in anorexia nervosa.* Appetite, 2010. **55**(2): p. 214-8.

14. Anderson, L.M., S.J. Crow, and C.B. Peterson, *The impact of meal consumption on emotion among individuals with eating disorders.* Eat Weight Disord, 2014. **19**(3): p. 347-54.

15. Holmes, A.P., R.C. Blair, J.D. Watson, and I. Ford, *Nonparametric analysis of statistic images from functional mapping experiments.* J Cereb Blood Flow Metab, 1996. **16**(1): p. 7-22.
